# TBX2 Expression predicts Tumor Recurrence and Adjuvant Chemotherapy Benefits in Gastric Cancer Patients following R0 Resection: a proposed approach for risk stratification

**DOI:** 10.7150/jca.34929

**Published:** 2020-03-04

**Authors:** Jun Lu, Yu Xu, Yao-hui Wang, Xiao-yan Huang, Yuan Wu, Jian-wei Xie, Jia-bin Wang, Jian-xian Lin, Ping Li, Chao-hui Zheng, Ai-min Huang, Chang-ming Huang

**Affiliations:** 1 Department of Gastric Surgery, Fujian Medical University Union Hospital, Fuzhou, China.; 2 Department of General Surgery, Fujian Medical University Union Hospital, Fuzhou, China.; 3 Key Laboratory of Ministry of Education of Gastrointestinal Cancer, Fujian Medical University, Fuzhou, China.; 4 Department of Pathology, the School of Basic Medical Sciences, Fujian Medical University.; 5 Institute of Oncology of Fujian Medical University, Fuzhou, China.; Jun Lu and Yu Xu contributed equally to this work and should be considered co-first authors

**Keywords:** TBX2, gastric cancer, survival, adjuvant chemotherapy

## Abstract

**Aims**: TBX2 is related to tumor progression and drug resistance. However, the roles of TBX2 in gastric cancer (GC) remain unclear. Our study aims at investigating the clinical roles of TBX2 in GC.

**Methods**: The protein expression levels of TBX2 in fresh GC tissue (n=20) were investigated with Western blotting analyses. The correlation between TBX2 expression and its prognostic significance was evaluated by immunohistochemical analyses of 401 patients. The survival benefit of postoperative adjuvant chemotherapy (PAC) for patients was evaluated.

**Results**: The expression of TBX2 was increased in GC tissue compared with adjacent paracancerous tissue (p=0.020). Immunohistochemistry demonstrated that TBX2 expression was significantly associated with lymphovascular invasion (p=0.024) and lymph node metastasis (p=0.044). A high level of TBX2 expression was an independent indicator of unfavorable recurrence-free and overall survival (p=0.002 and p=0.033, respectively). The prognostic model incorporating TBX2 expression exhibited greater predictive accuracy than the primary model. More importantly, the benefit of PAC noted in stage II/III GC patients with low TBX2 expression was superior to high TBX2 expression.

**Conclusion**: TBX2 may be not only a useful prognostic marker for GC but also a predictive biomarker of response to PAC in stage II/III GC patients. The current findings warrant further verification.

## Introduction

Gastric cancer (GC) is the fifth most common malignancy and the second leading cause of cancer-related deaths worldwide and is considered a major public health problem 1,2. At present, surgical resection is the only way to cure patients with GC; however, surgical outcomes remain unsatisfactory 3.

5-FU-based postoperative adjuvant chemotherapy (PAC) has been generally recommended as a first-line postoperative treatment for patients with stage II or stage III GC 4-7. Nonetheless, the therapeutic efficacy of PAC differs among individuals. One important explanation for this phenomenon is that the existing models for GC prognostic risk stratification and treatment strategy are predominantly established on the basis of tumor cell-oriented stratification systems, such as the TNM stage groupings. However, the TNM system has limitations and may not be sufficiently accurate when used alone in clinical circumstances since GC is a complex, heterogeneous disease with a highly variable prognosis 8. As a result, clinicians currently have little evidence to use when determining whether PAC will be beneficial to their patients 9. Consequently, there is a need to explore biomarkers for individualized risk stratification of the response to PAC in patients with GC.

T-box 2 (TBX2) is a member of the T-box family of transcription factors, which plays a critical role in embryonic development 10. In addition, recent studies report that TBX2 expression has been implicated in human cancer. TBX2 is overexpressed in breast 11, melanoma 12, colorectal 13 and pancreatic cancers 14. Importantly, Wansleben et al. 15 found that overexpression of TBX2 was also linked to chemotherapeutic drug resistance and that targeting TBX2 could improve the efficacy of current anticancer treatments. The findings of Nandana et al. 16 indicated that TBX2 acts as a novel therapeutic target for the treatment of metastasis in prostate cancer patients. Wang et al. 17 found a significant correlation between high TBX2 expression levels in primary tumors and reduced metastasis-free survival in breast cancer patients. Taken together, these results suggest that TBX2 may be an attractive new target for advanced stage cancer therapy. However, TBX2 expression and its predictive significance with respect to PAC in GC are not well understood. To date, only one study has reported the effect of TBX2 on overall survival for GC 18.

The aim of this study was to investigate the role of TBX2 in GC. Our results may not only shed light on the expression of TBX2 in GC tissue but also first pave the way for a promising prognostic system that can precisely evaluate the outcomes for GC patients and identify those who would benefit from receiving PAC.

## Materials and Methods

### Patients and clinical databases

Between January 1, 2015, and April 1, 2016, a total of 438 patients admitted to Fujian Medical University Union Hospital (FMUUH) were recruited for a randomized clinical trial with 419 patients included in the final analyses (ClinicalTrials.gov number NCT02327481). Details about the inclusion, exclusion, quality control and randomization have been previously reported 19. The selection criteria are as previously described. The present study is a sub-study of the above clinical trial. Patients who had neuroendocrine carcinoma or who underwent palliative surgery or neoadjuvant chemotherapy were excluded. After exclusion, the present analysis was restricted to 401 patients for whom curative gastrectomies were performed and for whom postoperative pathology confirmed stage I, II, or III gastric adenocarcinoma (pT1-4aN0-3M0) according to the 7th American Joint Committee on Cancer Staging system 20. Patients diagnosed at stage II or stage III were candidates to receive PAC. Collectively, 85.0% (226/266) of patients received 5-FU-based PAC in the stage II/III cohort (at least 1 cycle) 21. In addition, 20 fresh samples of GC and adjacent noncancerous tissue were obtained between June 1, 2018, and August 1, 2018, at FMUUH and used for Western blot analysis to determine the TBX2 expression levels. In this study, no patients had any treatment before surgery.

The study protocol was approved by the institutional review board of FMUUH, and the study was carried out according to the approved guidelines. Each subject was well informed about the details of this study, and informed consent was obtained. Recurrence-free survival (RFS) was defined as the time from surgery to the date of recurrence. Overall survival (OS) was calculated from the date of surgery to the date of death or last contact. The median follow-up for the entire cohort was 25 months (range 3-39 months).

### Western blot assay

20 fresh samples of GC and paired adjacent non-cancerous tissues were extracted with RIPA lysis solution (Thermo Fisher Scientific, Waltham, MA, United States) containing a PMSF (Roche, South San Francisco, CA, United States) with concentrations maintained at 1mM. Protein samples (40 μg per lane) were separated on 10%, polyacrylamide gels by the SDSPAGE method and transferred to PVDF membranes. Then, at room temperature, 5% skim milk was used to block the PVDF membrane for 1 h. The membrane was then incubated at 4 °C with the primary anti-TBX2 (ab157203, 1:1000 dilution; Abcam), or anti-GAPDH (ab8245, 1:2500 dilution; Abcam) and washed with TBS-T 3 times, 5 min each time, then incubated at room temperature with the HRP secondary antibody (Cell Signaling Technology) for 1 h. GAPDH was used as an internal control. Finally, the membrane was washed with TBS-T for 30 min and the protein bands were detected through an enhanced chemiluminescence method (Amersham Corporation, Arlin-gton Heights, IL, United States).

### Immunohistochemistry

A conventional immunohistochemical (IHC) staining protocol was used in this study. Briefly, paraffin-embedded tumor tissue blocks were cut into 4-μm-thick sections; then dried, deparaffinized, and dehydrated in a graded series of ethanol. Tissue sections were treated with 1% hydrogen peroxide for 10 min to block endogenous tissue peroxidase activity, followed by treatment with bovine serum for 30 min to reduce nonspecific binding. Antigen retrieval was then accomplished using citrate buffer (pH 6.0) with high-heat microwave processing for 5 min followed by low-heat microwave processing for 20 min. Immunohistochemistry was performed using rabbit anti-TBX2 antibody (1:300, MAIXIN BIO, Fuzhou, China). Slides were rinsed with phosphate-buffered saline before color development using a 3,3'-diaminobenzidine substrate kit and then counterstained with hematoxylin.

A semiquantitative immunohistochemistry score (IHS) was determined by evaluating both staining intensity (0, no staining; 1, weak staining; 2, moderate staining; 3, strong staining) and percentage of positive cells (1, 0% to 10%; 2, 11% to 50%; 3, 51% to 80%; and 4, 81% to 100%). The IHS was generated by multiplying the score of staining intensity and the score of the percentage of positive cells, which theoretically ranged from 0-12. According to previous reports 22,23, samples with HIS ≤ 4 were defined as low TBX2 expression, while those with TBX2 > 4 were considered as high expression. Two independent pathologists (Y.X and Y. W) who were blinded to the clinicopathological data and outcome of each patient evaluated the IHS. Statistical analysis was inspected by a third researcher who did not have a role in the scoring process.

### Statistical analysis

Associations between categorical variables were evaluated using Chi square tests. Kaplan-Meier method with log-rank tests was used for univariate survival analysis. Potential risk factors determined by univariate analysis were entered into multivariate analysis. The Cox proportional hazards model was undertaken in multivariate analysis to assess the independent effect of the variables. Hazard ratios (HRs) and the 95% CIs of covariates were calculated. The accuracy of the prognostic models was evaluated by Harrell's concordance index (C-index), Akaike information criterion (AIC), and receiver operating characteristic (ROC) curve analysis. Statistical analyses were performed using SPSS 19.0 (SPSS Inc, Chicago, IL) and R version 3.1.2 (R Foundation for Statistical Computing, Vienna, Austria). Statistical significance was set at 2-sided p <0.05.

## Results

### TBX2 expression in 20 pairs of fresh tissues evaluated by Western blotting

**Figure 1:**
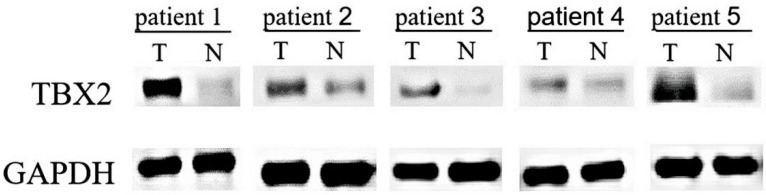
Western blot analysis of 5 representative paired tissue samples of gastric cancer (T) and their matched adjacent non-cancerous tissues (N).

The expression of TBX2 protein in the GC tissue and adjacent tissue was detected by Western blot analysis of 20 pairs of GC tissue and their adjacent tissue. Representative results are shown in Fig. 1. There were 15 patients (75%) whose GC tissue exhibited higher protein levels of TBX2 compared with those of the adjacent tissue. The results indicated that the expression of TBX2 protein in GC tissue was significantly higher than that in adjacent tissues (p=0.033).

### Location and expression of TBX2 protein in 401 GC patients by immunohistochemistry

**Figure 2:**
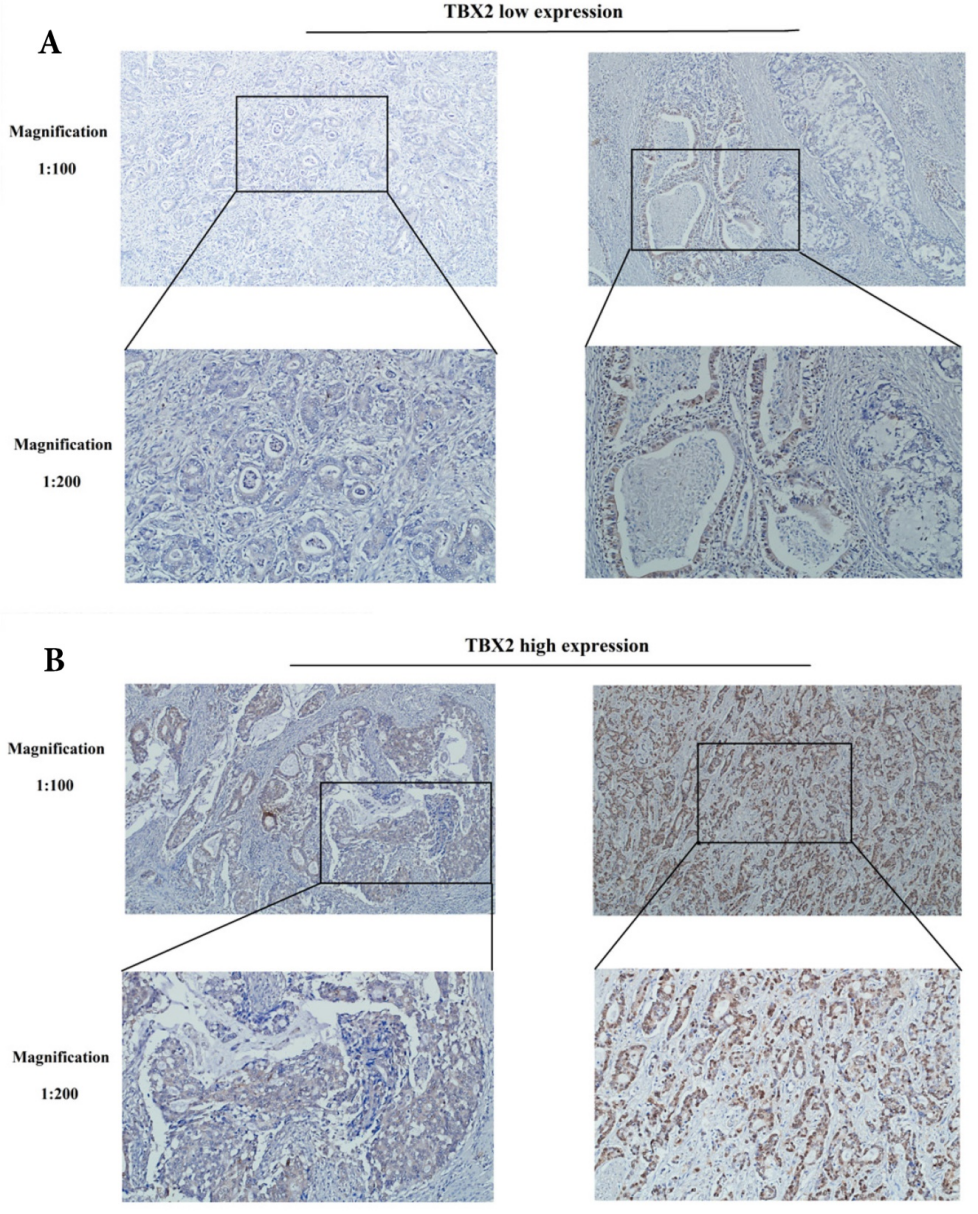
Representative photographs of **(A)** low and, **(B)** high expression levels of TBX2 in sections of gastric cancer.

Immunohistochemical analyses were performed to determine the location and expression of TBX2 protein in the GC tissue. Positive staining of the TBX2 protein was mainly located in the nuclei of tumor cells. Examples of TBX2-low and TBX2-high samples are shown in Fig. 2A and 2B, respectively. High expression of TBX2 was observed in 58.6% (235/401) of the GC samples, whereas 41.4% (166/401) of the GC samples had low expression of TBX2 (Table S1).

### Correlations between TBX2 protein expression and the clinicopathological features of GC patients

TBX2 expression and its relationships with the clinicopathological parameters of GC patients are listed in Table S1. Higher expression of TBX2 was significantly associated with lymphovascular invasion (p=0.024) and lymph node metastasis (p=0.044). These results indicated that TBX2 expression was associated with aggressive GC. However, no significant associations were found between TBX2 expression and sex, age, tumor location, tumor size, tumor differentiation, tumor depth or pathological TNM stage.

### High TBX2 protein expression is correlated with worse prognosis in GC patients

**Figure 3:**
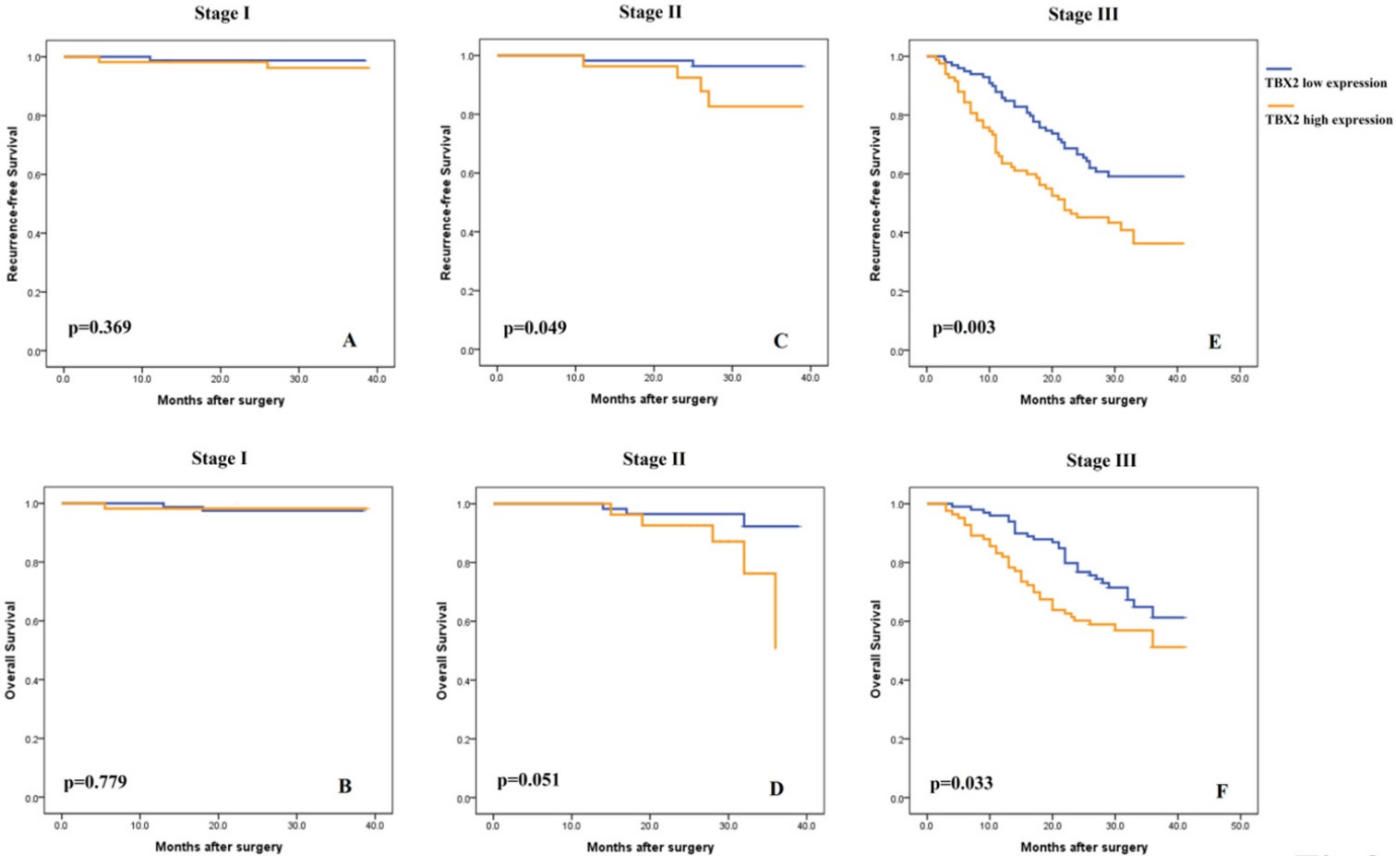
Subgroup Kaplan-Meier analysis of recurrence-free **(A,C,E)** and overall **(B,D,F)** survival in patients with resected gastric cancer according to TBX2 expression and tumor stage.

Subsequently, univariate analyses were conducted to evaluate the relationship of TBX2 protein expression and clinicopathological factors with the prognosis of GC patients. The results showed that a high expression level of TBX2 was significantly associated with poor RFS and OS of GC patients (p<0.001 and p=0.005, respectively) (Fig. S1), particularly for stage II (p<0.049 and p=0.051, respectively) and stage III (p<0.003 and p=0.033, respectively) patients (Fig. 3). After univariate analysis, multivariate analysis was conducted. Lymphovascular involvement (hazard ratio [HR]: 2.259, 95% confidence interval [CI]: 1.349-3.785, p=0.002), pathological TNM stage (HR: 5.310, 95% CI: 3.052-9.236, p<0.001), adjuvant chemotherapy status (HR: 0.509, 95% CI: 0.304-0.853, p=0.010) and TBX2-positive status (HR: 1.930, 95% CI: 1.284-2.900, p=0.002) were found to be independent prognostic markers for 3-year RFS. (Table S2). Similar independent prognostic factors were found for OS. (Table S3).

### Extension of prognostic models with TBX2 expression to patients with GC

To additionally evaluate the prognostic ability of TBX2 expression as a prognostic marker, we created prognostic models that combined TBX2 expression with TNM stage. As shown in Table 1, the C-indice of RFS was 0.7699 when evaluated with TNM stage alone, and it was enhanced to 0.8003 when the dichotomous TBX2 expression signature was placed in the combination model. Similarly, the C-index of OS was enhanced from 0.7428 to 0.7922 when the dichotomous TBX2 signature was placed into the model. Moreover, p values indicating the statistical significance of C-indices of the amalgamated TNM+ TBX2 model vs. the TNM model were all < 0.001 in RFS and OS analyses. AIC and ROC curve analyses also showed promising levels of predictive significance for TBX2 expression combined with TNM stage in GC patients (Table 1, Fig. S2).

### TBX2 expression and the benefit from PAC in stage II/III GC patients

**Figure 4:**
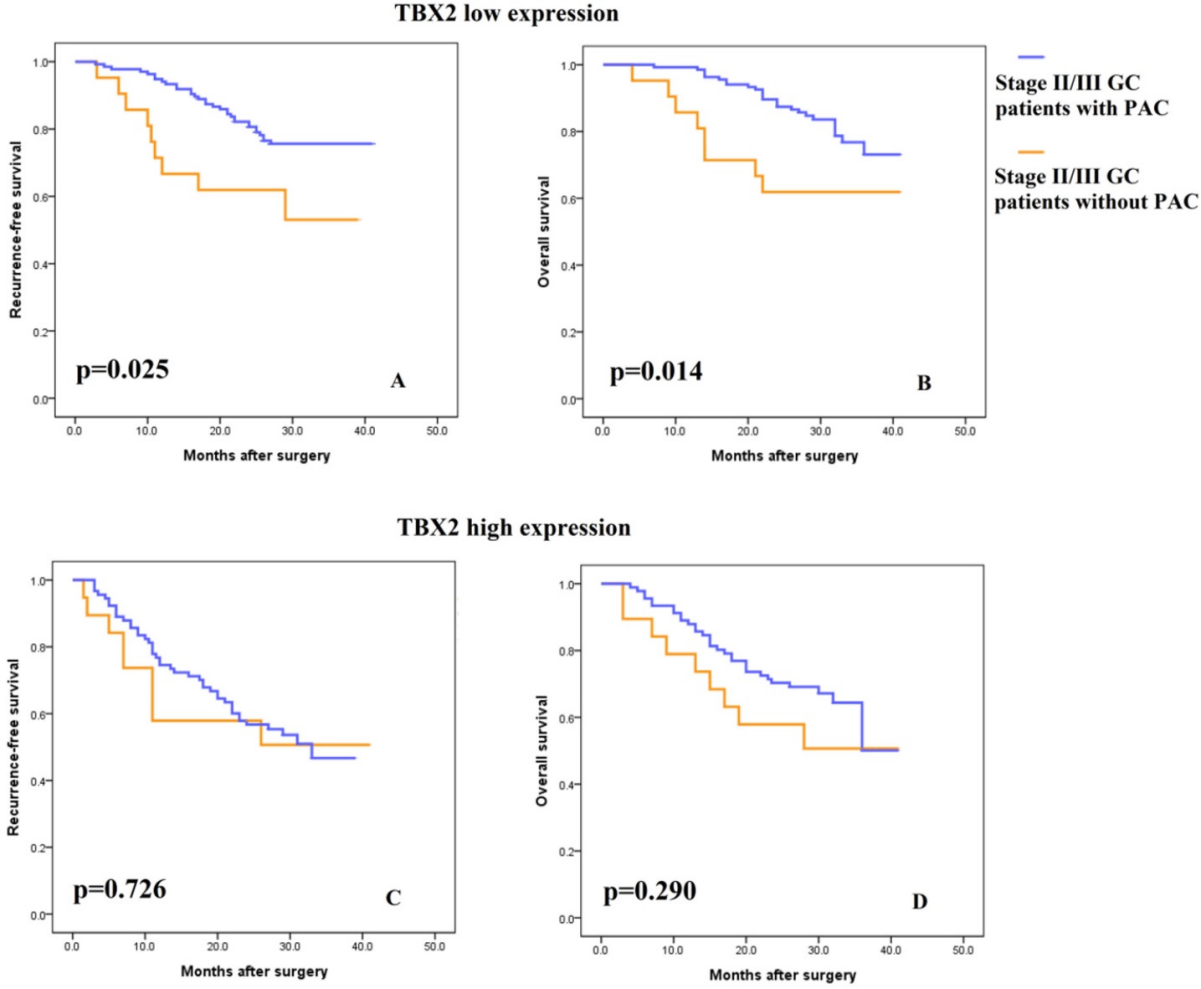
** (A-B)** Kaplan-Meier analysis of recurrence-free and overall survival in stage II/III patients with low TBX2 expression according to postsurgical adjuvant chemotherapy. **(C-D)** Kaplan-Meier analysis of recurrence-free and overall survival in stage II/III patients with high TBX2 expression according to postsurgical adjuvant chemotherapy.

Consistent with previous studies, 5-FU-based PAC was recommended for patients with stage II or III tumors. As shown in Fig. S3, PAC improved the survival rate of stage II/III patients. To explore the association of the TBX2 expression signature with the response to 5-FU-based PAC, subgroup analysis was performed in these patients. Incorporating the TBX2 signature into the PAC outcome data showed that, in the low TBX2 expression subgroup, patients with PAC had greater RFS and OS benefit than patients without PAC (Fig. 4A-B, log rank test p =0.025 and p=0.014, respectively). In contrast, in the high TBX2 expression subgroup, RFS and OS were as poor in the patients with PAC as in those without PAC (Fig. 4C-D, log rank test p=0.726 and p=0.290, respectively).

## Discussion

In this study, we showed that high TBX2 expression was positively associated with lymphovascular invasion, lymph node metastasis, and an increased risk of poor RFS and OS in GC. Furthermore, we expanded the prognostic model by including TBX2 expression and showed that the new model had better predictive accuracy than the TNM staging system. More importantly, we are the first to demonstrate that patients with low TBX2 expression might benefit from adjuvant chemotherapy.

Of the constructed GC prognostic models, the American Joint Committee on Cancer (AJCC) TNM staging system 20, which has been properly confirmed, is the most broadly utilized prognostic model to forecast outcomes in patients treated with radical operation. Nevertheless, a large weakness of this system is the difficulty in integrating new clinical information, including molecular markers or more elaborate bioinformatics 21.

Despite recent developments in therapy, the prognosis of advanced GC patients remains poor 24. PAC has been demonstrated to prolong stage II/III GC patients' survival periods. However, the lack of a reliable standard to identify those truly at high risk has made it hard for us to identify patients who would experience an RFS or OS benefit from adjuvant chemotherapy. In addition, the side effects of cytotoxic chemotherapy drugs can result in a decline in the quality of life when the patient is not sensitive to the chemotherapy. Thus, it is vital to distinguish patients who benefit from PAC from those who do not, and it would be helpful to have predictive biomarkers for chemosensitivity and chemoresistance to develop individualized therapy regimens.

Increasing evidence has established that TBX2 plays a role in the progression of a number of cancers 11-14,16. Han et al. 13 reported that TBX2 was a significant prognostic factor for decreased survival and increased disease recurrence independent of tumor stage and functioned as a marker to predict the prognosis of patients with colorectal cancer. Kandimalla et al. 25 recognized TBX2 as a pTa-specific prognostic biomarker in bladder cancer. Nandana et al. found that increased expression of TBX2 promotes bone metastasis 16. More importantly, the potential for using TBX2 as a target in cancer therapy is now being explored 13,17. TBX2 overexpression has been linked to chemotherapeutic drug resistance by an as yet unknown mechanism 15. Therefore, we believe that these findings reinforce the position of the current study that TBX2 might be used as a biomarker to predict prognosis and PAC benefit in patients with GC. We therefore examined the indicative prognostic and PAC selection values of TBX2 in GC.

Although Yu et al. 18 reported in their study that TBX2 is a prognostic factor for gastric cancer patients, the relationship between TBX2 expression and relapse-free survival or PAC benefit in gastric cancer has not been reported so far. In this study, we found a negative correlation between the TBX2 expression level and GC outcome and validated TBX2 as an independent prognostic marker for both RFS and OS in GC patients. Our evaluation further indicated that the integration of TBX2 into TNM staging might improve the risk stratification of GC patients. Importantly, we found that between stage II/III patients who received or did not receive PAC, the former group had better survival. When the TBX2 signature was incorporated, patients in the subgroup with low TBX2 expression who received PAC had longer survival than patients who did not receive PAC; however, patients with high TBX2 expression had no benefit from PAC, indicating that TBX2 expression could be an important factor for the efficiency of PAC. The findings can thus assist in choosing and treating patients who will be administered PAC. Based on the results of this study, among patients who were diagnosed with TNM II/III stage tumors, only those with low TBX2 expression were able to benefit from PAC, which makes TBX2 expression seem promising for stratifying patients more precisely.

A major limitation of this study was its retrospective design, although this was partially mitigated by using the prospective management database. Second, it was performed in a single center without external validation. Third, the underlying mechanism of cancer progression and drug resistance induced by TBX2 expression in GC has not been elucidated. Therefore, further experiments and validation in prospective cohorts or clinical trials are needed.

In conclusion, the findings indicated that TBX2 tumor cell expression could be identified as a novel prognostic predictor in GC clinical treatment, which may improve the current TNM system in terms of patient counselling. In addition, high TBX2 expression may use to define a subgroup of stage II/III gastric cancer patients who will be more likely to benefit from 5-FU-based PAC. Further studies into the mechanisms of TBX2 are thus warranted.

## Supplementary Material

Supplementary figures and tables.

## Figures and Tables

**Table 1:** Comparison of the Prognostic Accuracies of TNM Staging System and high TBX2 expression

	Model		p value
RFS	TNM	TNM+TBX2	
C-index	0.7699(0.7248-0.8209)	0.8003(0.7573-0.8359)	< 0.001
AUC (95% CI)	0.807(0.763-0.851)	0.837(0.797-0.878)	0.004
AIC	1009.875	1003.121	/
OS			
C-index	0.7428(0.6978-0.7968)	0.7922(0.7342-0.8218)	< 0.001
AUC (95% CI)	0.766(0.715-0.817)	0.791(0.741-0.840)	0.025
AIC	825.9906	819.1917	/

C-index indicates Harrell concordance index; AIC indicates Akaike Information Criterion; AUC indicates area under the curve.

## References

[1] Chen W, Zheng R, Baade PD, Zhang S, Zeng H, Bray F (2016). Cancer Statistics in China, 2015. CA Cancer J Clin.

[2] Ferlay J, Soerjomataram I, Dikshit R, Eser S, Mathers C, Rebelo M (2015). Cancer Incidence and Mortality Worldwide: Sources, Methods and Major Patterns in GLOBOCAN 2012. Int J Cancer.

[3] Okines AF, Thompson LC, Cunningham D, Wotherspoon A, Reis-Filho JS, Langley RE (2013). Effect of HER2 On Prognosis and Benefit From Peri-Operative Chemotherapy in Early Oesophago-Gastric Adenocarcinoma in the MAGIC Trial. Ann Oncol.

[4] Sakuramoto S, Sasako M, Yamaguchi T, Kinoshita T, Fujii M, Nashimoto A (2007). Adjuvant Chemotherapy for Gastric Cancer with S-1, an Oral Fluoropyrimidine. N Engl J Med.

[5] Sano T, Sasako M, Yamamoto S, Nashimoto A, Kurita A, Hiratsuka M (2004). Gastric Cancer Surgery: Morbidity and Mortality Results From a Prospective Randomized Controlled Trial Comparing D2 and Extended Para-Aortic lymphadenectomy-Japan Clinical Oncology Group Study 9501. J Clin Oncol.

[6] Sasako M, Sakuramoto S, Katai H, Kinoshita T, Furukawa H, Yamaguchi T (2011). Five-Year Outcomes of a Randomized Phase III Trial Comparing Adjuvant Chemotherapy with S-1 Versus Surgery Alone in Stage II Or III Gastric Cancer. J Clin Oncol.

[7] Jiang Y, Li T, Liang X, Hu Y, Huang L, Liao Z (2017). Association of Adjuvant Chemotherapy with Survival in Patients with Stage II or III Gastric Cancer. JAMA Surg.

[8] Cancer Genome Atlas Research Network (2014). Comprehensive Molecular Characterization of Gastric Adenocarcinoma. Nature.

[9] Lim L, Michael M, Mann GB, Leong T (2005). Adjuvant Therapy in Gastric Cancer. J Clin Oncol.

[10] Lu J, Li XP, Dong Q, Kung HF, He ML (2010). TBX2 and TBX3: The Special Value for Anticancer Drug Targets. Biochim Biophys Acta.

[11] Jacobs JJ, Keblusek P, Robanus-Maandag E, Kristel P, Lingbeek M, Nederlof PM (2000). Senescence Bypass Screen Identifies TBX2, Which Represses Cdkn2a (p19(ARF)) and is Amplified in a Subset of Human Breast Cancers. Nat Genet.

[12] Vance KW, Carreira S, Brosch G, Goding CR (2005). Tbx2 is Overexpressed and Plays an Important Role in Maintaining Proliferation and Suppression of Senescence in Melanomas. Cancer Res.

[13] Han Y, Tu WW, Wen YG, Yan DW, Qiu GQ, Peng ZH (2013). Increased Expression of TBX2 is a Novel Independent Prognostic Biomarker of a Worse Outcome in Colorectal Cancer Patients After Curative Surgery and a Potential Therapeutic Target. Med Oncol.

[14] Mahlamaki EH, Barlund M, Tanner M, Gorunova L, Höglund M, Karhu R (2002). Frequent Amplification of 8Q24, 11Q, 17Q, and 20Q-Specific Genes in Pancreatic Cancer. Genes Chromosomes Cancer.

[15] Wansleben S, Davis E, Peres J, Prince S (2013). A Novel Role for the Anti-Senescence Factor TBX2 in DNA Repair and Cisplatin Resistance.

[16] Nandana S, Tripathi M, Duan P, Chu CY, Mishra R, Liu C (2017). Bone Metastasis of Prostate Cancer Can be Therapeutically Targeted at the TBX2-WNT Signaling Axis. Cancer Res.

[17] Wang B, Lindley LE, Fernandez-Vega V, Rieger ME, Sims AH, Briegel KJ (2012). The T Box Transcription Factor TBX2 Promotes Epithelial-Mesenchymal Transition and Invasion of Normal and Malignant Breast Epithelial Cells. Plos One.

[18] Yu H, Liu BO, Liu A, Li K, Zhao H (2015). T-Box 2 Expression Predicts Poor Prognosis in Gastric Cancer. Oncol Lett.

[19] Zheng CH, Lu J, Zheng HL, Li P, Xie JW, Wang JB (2018). Comparison of 3D Laparoscopic Gastrectomy with a 2D Procedure for Gastric Cancer: A Phase 3 Randomized Controlled Trial. Surgery.

[20] Washington K (2010). 7Th Edition of the AJCC Cancer Staging Manual: Stomach. Ann Surg Oncol.

[21] Wu S, He H, Liu H, Cao Y, Li R, Zhang H (2018). C-C Motif Chemokine 22 Predicts Postoperative Prognosis and Adjuvant Chemotherapeutic Benefits in Patients with Stage II/III Gastric Cancer. Oncoimmunology.

[22] Chen X, Shao W, Huang H, Feng X, Yao S, Ke H (2019). Overexpression of RCN1 Correlates with Poor Prognosis and Progression in Non-Small Cell Lung Cancer. Hum Pathol.

[23] Cui G, Cai F, Ding Z, Gao L (2019). MMP14 Predicts a Poor Prognosis in Patients with Colorectal Cancer. Hum Pathol.

[24] Park JY, von Karsa L, Herrero R (2014). Prevention Strategies for Gastric Cancer: A Global Perspective. Clin Endosc.

[25] Kandimalla R, van Tilborg AA, Kompier LC, Stumpel DJ, Stam RW, Bangma CH (2012). Genome-Wide Analysis of CpG Island Methylation in Bladder Cancer Identified TBX2, TBX3, GATA2, and ZIC4 as pTa-specific Prognostic Markers. Eur Urol.

