# Supplementary material for: TBX2 Expression predicts Tumor Recurrence and Adjuvant Chemotherapy Benefits in Gastric Cancer Patients following R0 Resection: a proposed approach for risk stratification
Source: J Cancer. 2020 Mar 4;11(11):3172–9. doi: 10.7150/jca.34929 (PMC7097955; doi:10.7150/jca.34929)
Supplement: Supplementary file 1 — Supplementary figures and tables. [file jcav11p3172s1.pdf]

Entire cohort

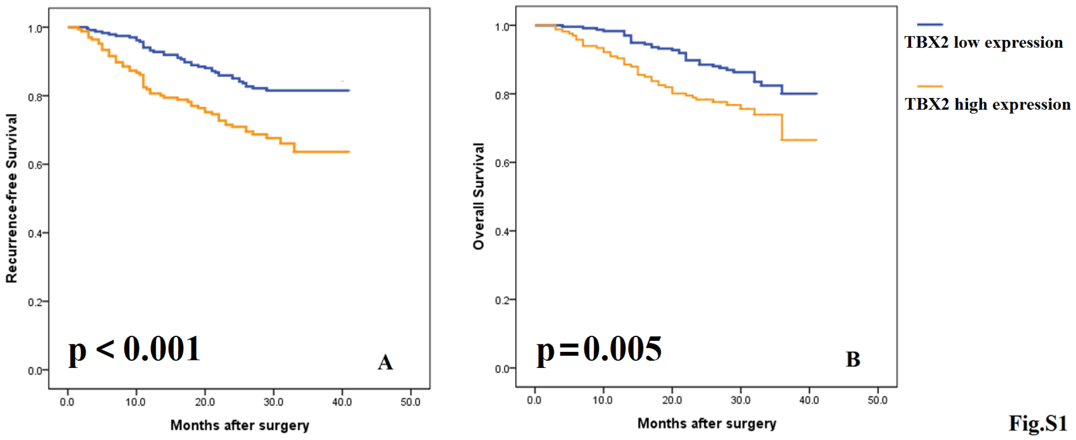

Fig.S1

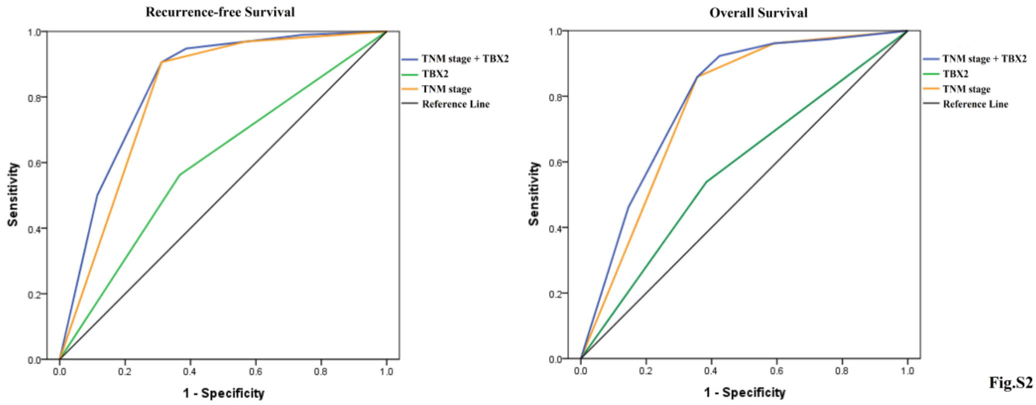

Fig.S2

Stage II/III patients

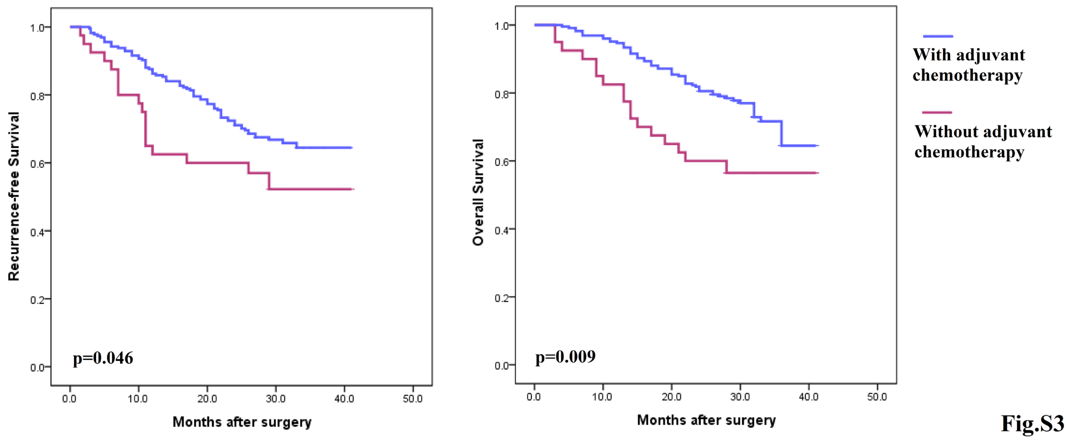

Fig.S3

Fig.S1 Kaplan–Meier analysis of recurrence-free (A) and overall (B) survival in patients with resected gastric cancer according to TBX2 expression.

Fig.S2 Receiver operating characteristic (ROC) curves for different prognostic models.

Fig.S3 Kaplan-Meier analysis of recurrence-free (A) and overall (B) survival in stage II/III patients according to whether received postsurgical adjuvant chemotherapy

Table S1 Correlation Between TBX2 and Clinicopathologic Characteristics

Characteristics	TBX2 Expression (n, %)		p value
	High	Low	
All cases	235 (58.6)	166 (41.4)	
Sex			0.542
Female	79 (33.6)	51 (30.7)	
Male	156 (66.4)	115 (69.3)	
Age (y)			0.191
＜65	166(70.6)	107(64.5)	
≥65	69(29.4)	59(35.5)	

Tumor location			0.974
Proximal	65 (27.7)	47(28.3)	
Middle	40(17.0)	29(17.5)	
Distal	107(45.5)	76(45.8)	
Entire	23(9.8)	14(8.4)	
Tumor size (cm)			0.768
＜5	138 (58.7)	100(60.2)	
≥5	97(41.3)	66(39.8)	
Differentiation			0.411
Differentiated	94(40.0)	74(44.6)	
Undifferentiated	141(60.0)	92(55.4)	
Lymphovascular invasion			0.024
Absent	117(49.8)	101 (60.8)	
Present	118 (51.2)	63(39.2)	
Tumor depth			0.183
T1/2	92 (39.3)	62(45.1)	
T3/4	143(60.7)	104(54.9)	
Lymph node metastasis			0.044
Absent	85 (36.1)	60(41.7)	
Present	150 (63.9)	106(58.3)	
Pathological stage			0.117
I	79(33.6)	56(33.7)	
II	38 (16.2)	40(24.7)	
III	118(50.2)	69(41.6)	
Adjuvant chemotherapy			0.207
Absent	79(33.6)	66(39.8)	
Present	156(66.4)	100(60.2)	

Table S2. Univariate and Multivariable Analysis of Recurrence-free Survival

	Univariate			Multivariable		
**Variables**	Hazard Ratio	95% CI	p value	Hazard Ratio	95% CI	p value
Sex (male)	1.246	0.779-1.945	0.332			
Age (≥65)	1.182	0.776-1.801	0.436			
Tumor location (lower third)	0.807	0.655-0.994	0.044	0.961	0.783-1.178	0.700
Tumor size (≥5cm)	3.792	2.480-5.800	<0.001	1.409	0.902-2.201	0.132
Tumor differentiation (undifferentiated)	2.130	1.357-3.341	0.001	1.289	0.854-1.579	0.897
Lymphovascular involvement	6.354	3.877-10.416	<0.001	2.259	1.349-3.785	0.002
Pathological stage (stage III vs I/II)	6.547	3.910-10.964	<0.001	5.310	3.052-9.236	<0.001
Adjuvant chemotherapy	0.435	0.266-0.712	0.001	0.509	0.304-0.853	0.010
TBX2 expression (high)	2.075	1.386-3.106	<0.001	1.930	1.284-2.900	0.002

Table S3. Univariate and Multivariable Analysis of Overall Survival

	Univariate			Multivariable		
**Variables**	Hazard Ratio	95% CI	p value	Hazard Ratio	95% CI	p value
Sex (male)	1.053	0.065-1.704	0.832			
Age (≥65)	1.396	0.884-2.203	0.152			
Tumor location (lower third)	0.924	0.738-1.156	0.488			
Tumor size (≥5cm)	3.581	2.236-5.736	<0.001	1.152	0.941-2.560	0.085
Tumor differentiation (undifferentiated)	2.115	1.282-3.489	0.003	1.081	0.643-1.820	0.768
Lymphatic involvement	4.438	2.774-7.100	<0.001	2.286	1.270-4.114	0.006

Pathological stage (III vs I/II)	6.368	3.624-11.189	＜0.001	3.399	1.989-5.808	＜0.001
Adjuvant chemotherapy	0.518	0.306-878	0.014	0.520	0.297-0.911	0.022
TBX2 expression (high)	1.862	1.193-2.908	0.006	1.633	1.041-2.560	0.033
